# The Cell Wall Hydrolytic NlpC/P60 Endopeptidases in Mycobacterial Cytokinesis: A Structural Perspective

**DOI:** 10.3390/cells8060609

**Published:** 2019-06-18

**Authors:** Flavia Squeglia, Miguel Moreira, Alessia Ruggiero, Rita Berisio

**Affiliations:** Institute of Biostructures and Bioimaging (IBB), CNR, 80134 Naples, Italy; flavia.squeglia@cnr.it (F.S.); miguel.moreira@ibb.cnr.it (M.M.); alessia.ruggiero@cnr.it (A.R.)

**Keywords:** cell division, mycobacteria, structure, peptidoglycan, NlpC/P60 endopeptidase, RipA

## Abstract

In preparation for division, bacteria replicate their DNA and segregate the newly formed chromosomes. A division septum then assembles between the chromosomes, and the mother cell splits into two identical daughters due to septum degradation. A major constituent of bacterial septa and of the whole cell wall is peptidoglycan (PGN), an essential cell wall polymer, formed by glycan chains of β−(1-4)-linked-*N*-acetylglucosamine (GlcNAc) and N-acetylmuramic acid (MurNAc), cross-linked by short peptide stems. Depending on the amino acid located at the third position of the peptide stem, PGN is classified as either Lys-type or meso-diaminopimelic acid (DAP)-type. Hydrolytic enzymes play a crucial role in the degradation of bacterial septa to split the cell wall material shared by adjacent daughter cells to promote their separation. In mycobacteria, a key PGN hydrolase, belonging to the NlpC/P60 endopeptidase family and denoted as RipA, is responsible for the degradation of septa, as the deletion of the gene encoding for this enzyme generates abnormal bacteria with multiple septa. This review provides an update of structural and functional data highlighting the central role of RipA in mycobacterial cytokinesis and the fine regulation of its catalytic activity, which involves multiple molecular partners.

## 1. Introduction to Cytokinesis in Bacteria

**Figure 1:**
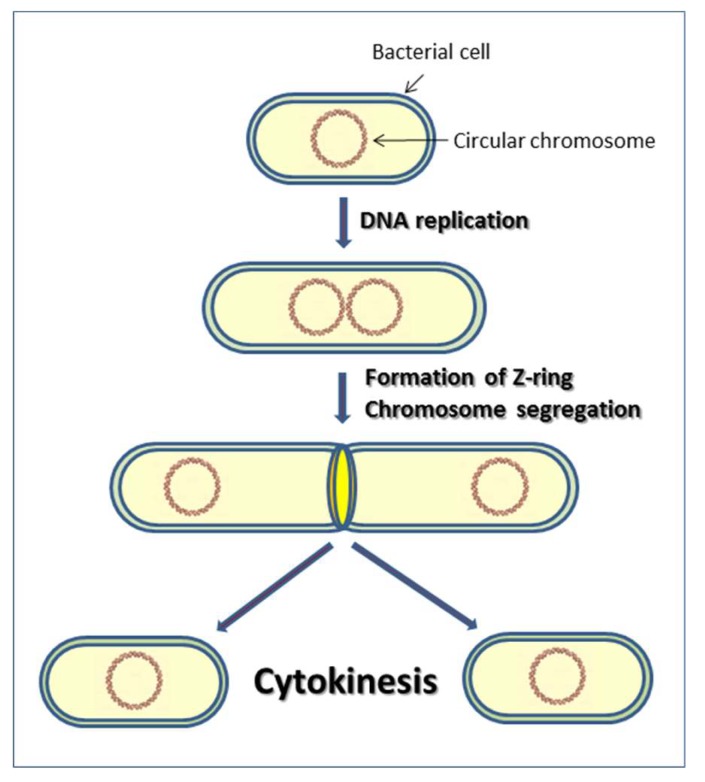
The cytokinesis process.

The bacterial cell division is a complex cell cycle by which a parent cell divides into two daughter cells. This process is characterized by two main events: chromosome segregation and cytokinesis (Figure 1) [1,2]. A single, circular chromosome replicates its DNA and, after replication, the two chromosomes move towards opposite ends of the cell in a process called segregation. After the completion of chromosome segregation, the division process begins with the formation of the septal ring, called Z-ring, a polymer of the tubulin-like GTPase FtsZ, which is almost universally conserved [3,4,5] (Figure 1). The Z-ring is stabilized by FtsA and ZipA (Z-interacting protein A) [6,7]. Subsequently, other protein factors are recruited to the cytokinetic ring, forming a piece of complex machinery called the divisome [8,9].

In *Mycobacterium tuberculosis*, FtsZ has been recognized as one of the major cytoskeletal organizers of the mycobacterial divisome [10]. Indeed, depletion of FtsZ results in long filamentous cells [4,10]. The mechanisms by which a Z-ring composed of FtsZ subunits contracts remains enigmatic. In *M. tuberculosis*, it has been proposed that GTP hydrolysis and/or nucleotide release induces a conformational change that alters the intersubunit packing of FtsZ polymer, an event which likely promotes ring contraction [11].

The ring formed by FtsZ recruits both structural and enzymatic proteins involved in peptidoglycan (PGN) synthesis and, thus, in the formation of the septum [12,13]. Then, this septal PGN, initially shared between daughter cells, must be degraded by PGN hydrolases to complete the division process. However, a central problem is to understand how the events of cell division inside the cell send signals that trigger PGN hydrolysis by hydrolases outside the cell. A currently accepted model is that FtsE and FtsX sense the progress of cell division and regulate extracellular PGN hydrolases. Of these, FtsE links the Z-ring to the transmembrane protein, FtsX, which senses the PGN hydrolases outside the cell [14]. More specifically, FtsE hydrolyzes ATP to ADP upon sensing an unknown signal from inside the cell. This hydrolysis causes a conformational change that is transmitted through the membrane via FtsX. A conformational change of the extracellular part of FtsX results in an interaction with either cell wall hydrolases or effector proteins and activation of PGN hydrolysis [15].

In *Escherichia coli*, FtsX controls the activity of the PGN amidases AmiA and AmiB through interactions with the effector protein EnvC [16], whereas in *Streptococcus pneumoniae*, FtsX is known to interact with PcsB, a putative CHAP (Cys, His, Asp peptidase) protein predicted to hydrolyze PGN cross-links [17]. In mycobacteria, several PGN hydrolases have been discovered in recent years, including (i) one N-acetylmuramoyl-L-alanine amidase [18,19]; (ii) five homologous endopeptidases of the NlpC/P60 family, among which RipA plays a central role [20,21,22]; and (iii) a set of five homologous glycosidases, named resuscitation-promoting factors, RpfA-E, which are known to be involved in mycobacterial resuscitation from dormancy [23,24,25]. This work will review structural and functional data related to PGN hydrolases involved in cell division and the complex mechanism of regulation of PGN hydrolysis in mycobacteria.

## 2. The Peptidoglycan Nature of The Septal Ring 

The cell envelope of the *M. tuberculosis* plays a key role in bacterial virulence and antibiotic resistance, although there is still a lot to discover about the molecular mechanisms of regulation of cell-envelope formation. Since it is unique among prokaryotes, its proteins, carbohydrate, and lipid components have been the subject of interest for developing new vaccines [26,27,28,29]. The molecular and architectural complexity of the mycobacterial cell wall is strictly related to the pathogenicity of the bacterium [30,31]. The uncommon impermeable properties of the *M. tuberculosis* cell wall and the richness in high molecular weight lipids provide a thick layer involved in *M. tuberculosis* resistance to antibiotics and stressful conditions [32]. 

**Figure 2:**
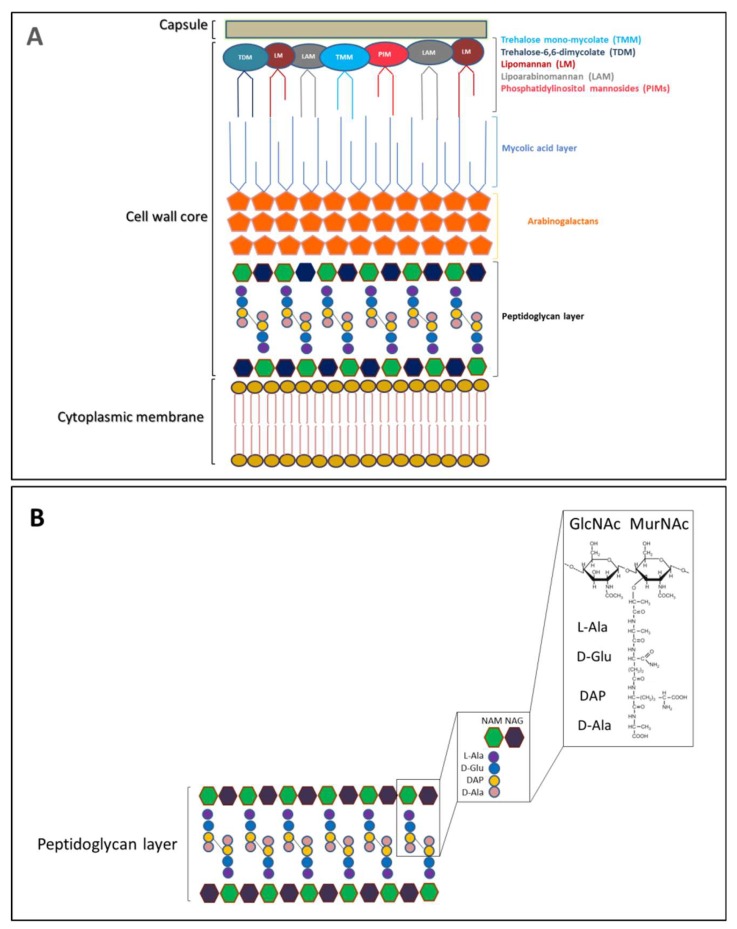
*Mycobacterium tuberculosis* cell envelope. (**A**) Overall architecture of the cell envelope of *Mycobacterium tuberculosis*. (**B**) A schematic representation of peptidoglycan (PGN) along with the chemical structure of a PGN monomer.

*M. tuberculosis* cell envelope is made up of three parts: the plasma membrane, the cell wall core, and the capsule. The cell wall core consists of two parts, named as lower and upper segments. The lower segment is composed of an impermeable layer of mycolic acids (MA) connected to a peptidoglycan layer through arabinogalactans (AG) (Figure 2A) [26]. This mycolyl-arabinogalactan-peptidoglycan (mAGP) complex is crucial for the viability of *M. tuberculosis* and it is important for cell wall integrity and osmotic stability [26]. The upper segment, also named as outer membrane, contains proteins, sulfolipids, phosphatidylinositol mannosides (PIMs), phthiocerol-containing lipids, lipomannan (LM), lipoarabinomannan (LAM), and mycolic acids esters, principally trehalose-6,6-dimycolate (TDM) and trehalose mono-mycolate (TMM) (Figure 2A) [27,28,29]. Several studies have shown that PIMs, LM, and LAM exhibit important and distinct immunomodulatory properties. Indeed, they regulate the production and secretion of pro-inflammatory cytokines during mycobacterial phagocytosis by macrophages [26,28,33]. The most cell-surface-exposed material is composed of capsular polysaccharides, proteins, and small amounts of lipids [34,35]. Surface proteins and polysaccharides are responsible for adhesion, penetration, infection, and survival of *M. tuberculosis* in the host cells [36,37,38,39,40]. 

PGN is an essential and dynamic element of the mycobacterial cell wall. Its biosynthesis is targeted by many potent antibiotics and several enzymes involved in cell division final process, due to its abundance at the bacterial septal ring [41,42,43]. It is a polymer composed of alternating *N*-acetylglucosamine (GlcNAc) and N-acetylmuramic acid (MurNAc) residues, linked in a β(1→4) configuration [44]. These glycan strands are cross-linked by short peptides containing peculiar amino acids, for example d-Ala, d-Glu and meso-diaminopimelic acid (DAP) (Figure 2B).

## 3. Septal Ring Degradation and Daughter Cell Division

Septal PGN is initially shared between daughter cells and must be degraded by PGN hydrolases to complete the division process. Bacterial cellular division ends with cell disconnection, a mechanism needed to disconnect the two new daughter *sacculi* after the cell division is completed, or at the very late step of cell division. The process of daughter cell separation requires a delicate balance of cell wall hydrolases that cleave the septa connecting the daughter cells. Cell-separating enzymes usually contain endopeptidase domains, like cysteine histidine aminopeptidase (CHAP) or NLPC/P60 domains, and/or glucosaminidase domains [45,46,47]. Interestingly, as many as 18 hydrolases are known to be involved in septum cleavage of *E. coli*, while only a few hydrolases are known in mycobacteria [41,42,48] (Table 1).

**Figure 3:**
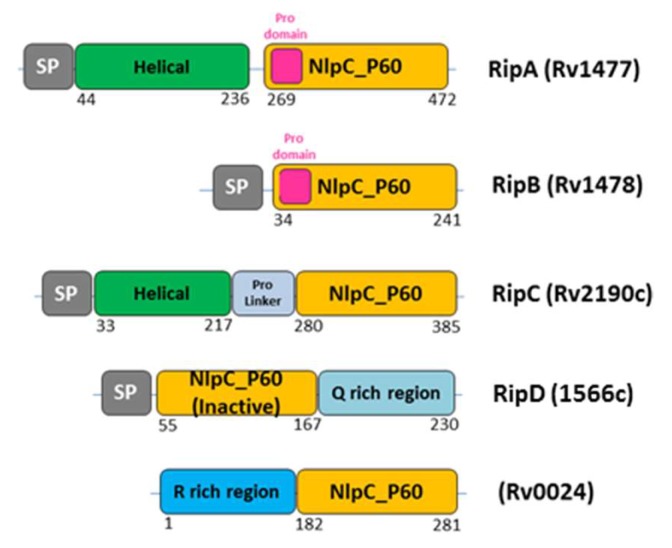
Schematic view of all five NlpC/P60 homologs encoded in the *M. tuberculosis* genome.

As described above, five NlpC/P60 proteins are predicted to be secreted by *M. tuberculosis*, although Rv0024 lacks a recognizable signal peptide (SP). RipA (Rv1477), RipB (Rv1478), RipC (Rv2190c), and Rv0024 show the typical architecture, with the catalytic domain at the C-terminus (Figure 3). Differently, RipD (Rv1566c) encodes an N-terminal catalytic domain. This domain does not show peptidoglycan hydrolase activity, which is consistent with the sequence alterations at the catalytic site [52]. RipA and RipB contain pro-domains just before the catalytic domain, which were shown to inactivate the enzymes [20,51]. In contrast, the RipC catalytic domain is preceded by a proline-rich linker with no inactivating function [57]. In addition, both RipA and RipC contain a predicted N-terminal coiled-coil domain (Figure 3).

RipA is the largest and more complex endopeptidase. It is a key enzyme for *M. tuberculosis* cell division, with remarkable effects on the bacterial phenotype, as shown in *M. smegmatis*. Indeed, RipA depletion strains exhibit a decreasing growth and an abnormal phenotype, consisting of branching and chaining bacteria [58]. Similar to other cell separating endopeptidases, like CwlT from *Bacillus subtilis* [59] and Spr from *E. coli* [60], RipA hydrolyzes peptidoglycan peptide crosslinks [21].

**Figure 4:**
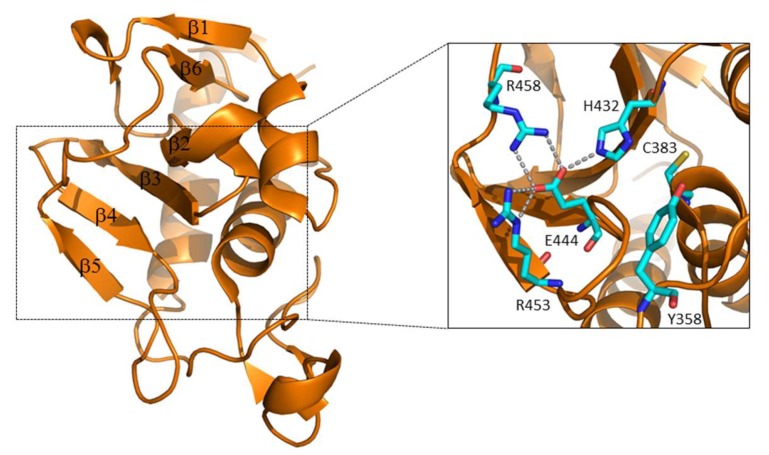
Cartoon representation of the RipA catalytic domain structure. The inset shows a zoom with key residues shown with ball-and-stick.

Crystallographic studies of RipA first [20] and then of RipB [51] have yielded new insights into the functional regulation of these enzymes. The structure of RipA catalytic domain comprises a central β-sheet of six antiparallel β-strands, a small two-stranded β-sheet, and six helices, arranged in an αββααββββββ topology (Figure 4). Its putative catalytic cysteine (Cys383) is located at the N-terminal end of a helix (α2) and is packed against the six-stranded β-sheet core (Figure 4). At this location, Cys383 facets another conserved residue, His432, belonging to the β-strand β3 (Figure 4). This histidine is hydrogen bonded to the side chain of Glu444, which is, in turn, tightly anchored to Arg453 and Arg458 (Figure 4). Notably, the crystal structure suggested a Cys-His-Glu catalytic triad, which is unusual considering the typical Cys-His-His triad of NlpC/P60 endopeptidases (Figure 4) [20]. 

**Figure 5:**
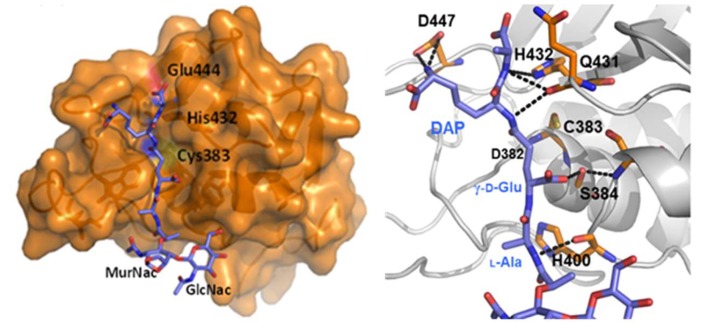
Modeling of a PGN monomer in the catalytic site cleft of RipA (left) and main interactions of the PGN monomer with the enzyme [49].

The composition of this triad was confirmed by mutational studies, as the mutation of each residue of the triad to alanine completely suppresses RipA activity [49]. As shown in Figure 5, the catalytic site cleft shape accounts well for the branched nature of peptidoglycan, whose monomer has the structure GlcNAc-MurNAc-L-Ala-γ-D-Glu-DAP-D-Ala. Aside from the catalytic triad, several residues interact with the muropeptide (D382, S384, S402, V428, Q431, D447) (Figure 5). Among these, the most conserved is D382, which directly contacts γ-D-Glu; this indicates a strong specificity of RipA towards γ-D-Glu [49]. Differently, scarce conservation characterizes residues contacting DAP side chain (e.g., D447), a finding that agrees well with the ability of RipA to hydrolyze both Lys-type and DAP-type PGN [20].

**Figure 6:**
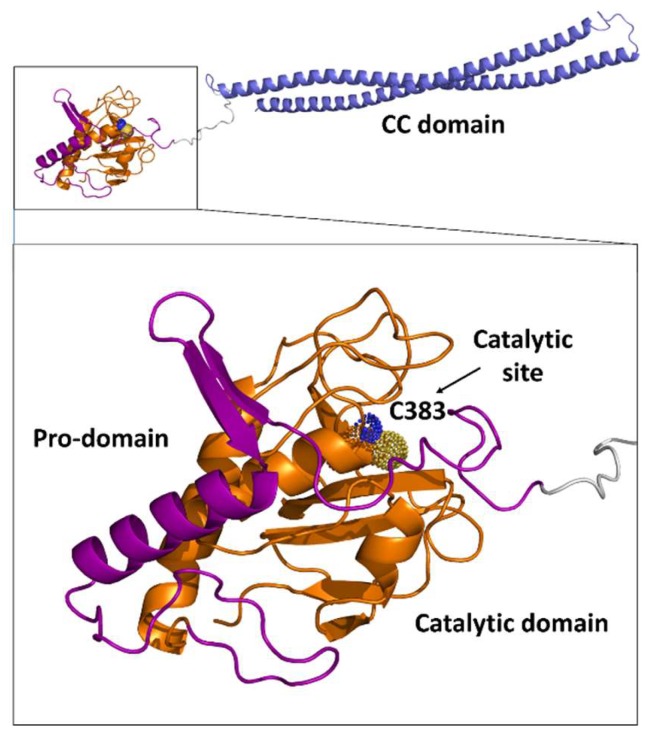
Cartoon representation of the entire structure of RipA: the catalytic and pro-domains (PDB code 3ne0) are shown in orange and purple, respectively. The catalytic C383 is highlighted in dot representation. The helical domain (PDB code 6ewy) is shown in blue; the linker is shown in grey.

**Figure 7:**
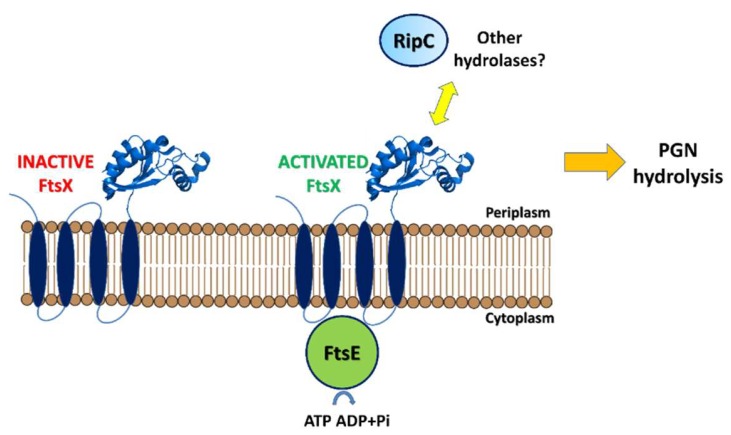
Model for the activation of hydrolases by FtsX. FtsE hydrolyzes ATP to ADP upon sensing an unknown signal from inside the cell. This hydrolysis causes a conformational change that is transmitted through the membrane via FtsX. A conformational change of the extracellular part of FtsX results in interaction with either cell wall hydrolases or effector proteins and activation of PGN hydrolysis.

Structural studies also showed that a tight array of interactions exist between the catalytic domain and the pro-domain [20]. In particular, the catalytic site cleft of the enzyme is physically blocked by the pro-domain, which contains a catalytic-cleft-blocking loop region, a β hairpin, and a long α helix, nearly perpendicular to the β hairpin (Figure 6). A tight interaction is observed between these two domains, with a total interaction surface area of 1809 Å^2^ [20]. This finding strongly suggested functional inactivity of the enzyme in this form and revealed a zymogenic nature for RipA. Consistently, cell wall degradation assays showed that activation of RipA requires the release of the pro-domain [20]. A strong structural conservation was observed for the catalytic domains of RipA and RipB, whereas structural differences characterized the pro-domains. In RipB, the pro-domain is folded in two helices wrapped around the catalytic domain, suggesting a similar regulatory function as observed for RipA [51]. Compared to RipB, RipA contains an extra N-terminal domain, whose structure has been recently determined [50] (PDB code 6ewy). This domain is formed by two helices of similar length, connected by a 6-residue loop, and forms a long-coiled coil structure (Figure 6). Whereas the pro-domain of RipA has a regulating function, its helical domain does not influence the accessibility of the active site (Figure 6). Instead, its rigid stalk-like module is typical of scaffold building proteins. Possibly, this non-catalytic domain is responsible for anchoring the enzyme to the divisome, although interactions of RipA with the divisome have so far not been demonstrated [50]. Different from RipA, the homologue NlpC/P60 endopeptidase RipC interacts with FtsX [57], and this interaction favors a long-range conformational change that activates RipC [15,57] (Figure 7). As observed for other bacteria, hydrolysis of ATP by FtsE is believed to cause a conformational change that is transmitted through the membrane via FtsX to the extracellular region [15]. In *M. tuberculosis*, the conformational change of the extracellular part of FtsX results in interaction with RipC, an event which causes RipC conformational activation [57] (Figure 7). It should be noted that RipC does not contain an inactivating pro-domain, different than RipA and RipB (Figure 3). Therefore, it is likely that its mechanism of activation differs from those of RipA and RipB. In these latter cases, the tight interaction of the catalytic sites with the pro-domains is in line with an alternative activation mechanism, which requires proteolytic activation [20,22]. Although studies are still to be undertaken, it is presumable, as demonstrated for RipC, that, in all cases, a coordinated mechanism exists that regulates Z-ring formation by FtsZ, septation, and septum degradation.

### 3.1. The Regulation of RipA Through MarP

**Figure 8:**
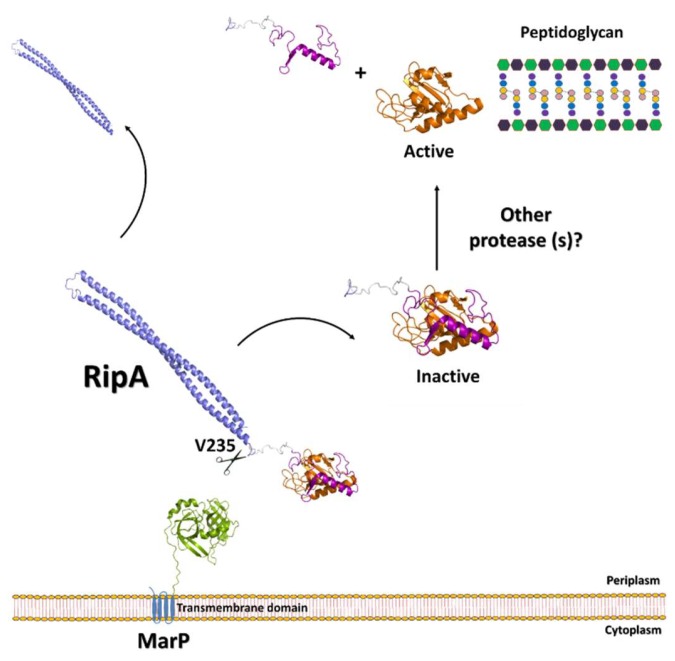
Regulation of RipA via proteolytic degradation by MarP. Given the site of hydrolysis, at position V235, a successive hydrolysis is needed to activate RipA.

As previously discussed, structural and biochemical studies have suggested that RipA needs to be proteolytically processed to achieve hydrolysis activity [20]. A proteolytic activation mechanism has been successively confirmed in vivo [22]. In addition, it has been shown that overproduction of activated RipA produces severe growth defects in *M. tuberculosis* [22]. More recently, RipA has been demonstrated to be the substrate of MarP (*Mycobacterium* acid resistance protease, Rv3671c), a serine protease which has been associated to the resistance of *M. tuberculosis* to the acidic conditions of phagolysosomes [61]. MarP is a highly conserved protein in mycobacteria, required for pH homeostasis and survival in a hostile environment [62,63]. Its N-terminal domain includes four putative transmembrane helices that likely anchor the protease to the cytoplasmic membrane [64]. The chymotrypsin-like serine protease domain of MarP is located at its C-terminus and contains the catalytic triad, composed of His235, Asp264, and Ser343, conserved in the serine protease family (PDB code 3k6y) [65]. With this topology, the C-terminal protease domain is placed in a periplasmic localization, essential to exert its hydrolytic function on RipA (Figure 8). Indeed, biochemical studies have demonstrated that these two proteins interact in vivo in acidic conditions [61].

Interestingly, *M. tuberculosis* cells that lack MarP are hypersensitive to acidic pH [66] and share similar phenotypes as cells lacking RipA. Indeed, both mutants display increased cell length and form chains in acidic conditions [61]. These data indicate that the inability of MarP-deficient cells to survive acidic stress depends on their inability to activate RipA, and that PGN hydrolysis is essential for *M. tuberculosis* survival in acidic conditions [61]. In these conditions, activation of RipA by MarP assures PGN remodeling, rearrangement, or repair, all steps which are likely vital to the survival of *M. tuberculosis* during acidic stress. It should be noted that the identified site of RipA hydrolysis by MarP is specifically located after Val235, which belongs to the N-terminal domain of RipA (Figure 8). This cleavage does not unlock the catalytic cleft of RipA, which remains occluded by the regulatory domain (Figure 8). However, the release of RipA from its stalk N-terminal domain likely exposes the enzyme to other proteases for the fine processing that activates RipA for PGN degradation. Also, it cannot be excluded that activation of RipA in non-acidic stress conditions may be regulated by other proteases. In addition, it is still to be established what the link is between septation through the assembly of the Z-ring and PGN hydrolysis by RipA, as no interactions between these two processes have yet to been proven.

### 3.2. Regulation of RipA Via Protein-Protein Interactions

**Figure 9:**
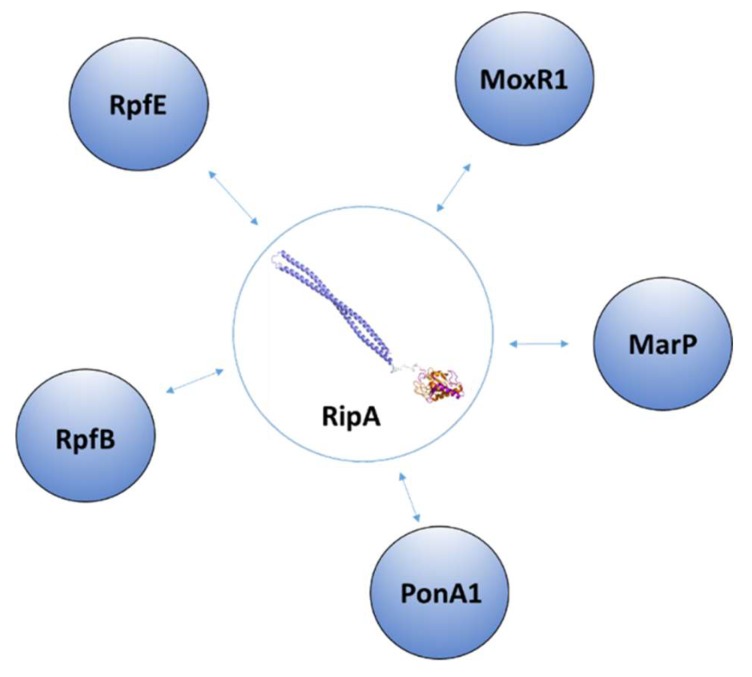
Interacting partners of RipA.

RipA was identified as a protein localized in the septum of dividing mycobacteria and was proposed to be part of the divisome [41]. RipA has a wide pattern of interactors (Figure 9). Besides being cleaved by MarP, RipA was shown to interact with a pool of PGN modeling enzymes, including the resuscitation-promoting factors RpfB (Rv1009) and RpfE (Rv2450c), and the penicillin-binding protein PonA1 (Rv0050) [67,68]. In addition, RipA is able to interact with MoxR1, a member of the ATPase family that is associated with various cellular activities. MoxR1 protein displays ATP-enhanced chaperone activity and MoxR1-mediated folding of RipA is critical for its secretion within the TAT pathway system [69].

Interestingly, a strong connection exists between cell division and resuscitation from the dormant state, characterized by low metabolic activity and resistance to antibiotics [70,71,72]. A latent infection, due to dormant bacteria, can develop into active disease even decades after initial infection, when the immune response weakens [73,74]. Since about one-third of the world’s population is infected with dormant *M. tuberculosis*, the risk of disease reactivation is troublesome. Resuscitation from the dormant state is attributed to a set of PGN hydrolases, denoted as resuscitation-promoting factors (Rpfs) [70,75,76,77,78,79,80]. RipA co-localizes at bacterial septa with the resuscitation-promoting factor RpfB [67], which exhibits the highest structural complexity among the five Rpfs. Whereas RpfA, RpfC, RpfD, and RpfE are mainly composed of a lysozyme-like catalytic domain, RpfB contains a further four domains: a G5 domain and three DUF348 domains, in addition to the catalytic domain. Structural studies of RpfB have been crucial to elucidate the functional features of this molecule [24,25,53,81].

Similar to lysozyme, the protein catalytic cleft consists of six carbohydrate-binding sites (A–F), thus, corroborating the hypothesis that RpfB acts by hydrolyzing the carbohydrate component of PGN [53]. Crystallographic studies have also evidenced a novel fold of the G5 domain, constituted by two β-sheets connected by a small triple helix motif, and denominated as β-TH-β [25], which has been connected to PGN adhesion [25]. Also, the structural description of the DUF348 domain revealed an unexpected structural similarity to eukaryotic ubiquitin, a small protein that exists in all eukaryotic cells both to target proteins for rapid degradation by the proteasome [24]. The presence of an ubiquitin-like domain in RpfB may be responsible for its ability to interact with several proteins, by sharing a strong feature of ubiquitin: its promiscuity due to its intrinsic high molecular adaptability [82]. PGN hydrolase activities of RipA and RpfB are synergic although the structural basis of this synergistic action is hitherto not clear [21], as it may either be associated with the allosteric activation of one of these proteins or to their enhanced ability to release free muropeptides by acting both on the glycan and peptide moieties [83]. Along this line, it has been shown that the joint action of RpfB and RipA on PGN produces a reaction product of particular chemical nature, anhydroGMDP, which directly induces resuscitation of the dormant cell cultures [83]. This finding also agrees well with the previous finding, showing that resuscitation from dormancy is a complex phenomenon, involving direct interaction of muropeptides released upon PGN hydrolysis with a set of STPK kinases [40,84,85,86,87,88,89]. Interestingly, the synergy between hydrolytic actions RipA and RpfB of PGN can be inhibited by the interaction of RipA with the penicillin-binding protein PonA1, a key PGN synthase, which also co-localizes at the poles and septa of dividing cells and is a central determinant of polar growth in mycobacteria [68,90]. This finding indicates universal molecular mechanisms that coordinate cell wall synthesis and degradation through protein-protein interactions between enzymes with antagonistic functions.

## 4. Concluding Remarks

As discussed in this review, cytokinesis is regulated in *M. tuberculosis* by a set of endopeptidases of the NlpC/P60 family. Similar to other cases of proteins responsible for key cell processes, a high redundancy characterizes these enzymes, since five endopeptidases are encoded in the mycobacterial genome. However, the reason for the existence of these redundant enzymes, sharing a similar structure, remains elusive. NlpC/P60 endopeptidases are potentially suicide enzymes since they are able to hydrolyze the mycobacterial cell wall. Consistently, their catalytic activities are strongly regulated. Among these NlpC/P60 endopeptidases, RipA plays a central role, as it is essential to cleave the mycobacterial PGN septum, which is synthesized right after chromosome segregation. The data accumulated in recent years concerning the main endopeptidases structure and function provided a more detailed picture of their catalytic and regulatory mechanisms. In RipA, catalytic regulation proceeds both through proteolytic cleavage and through the interaction with multiple molecular partners. Differently, activation of its homolog RipC proceeds through a conformational alteration due to interactions with the divisome protein FtsX. Going forward, it will be important to define in more detail which mechanisms correlate septal PGN formation following Z-ring formation with PGN degradation. 

Recent studies have shown that the use of hydrolases, such as RipA, alters the physiological state and induces stress responses in *M. smegmatis*, but it does not completely inhibit mycobacterial growth [91]. However, when used in conjugation with common antimicrobial drugs, such as rifabutin and bacitracin, RipA enhances their cytotoxic activities. This finding brings further advantages for drug development and antimicrobial treatment, as it highlights the potential for a new strategy that combines endopeptidases with previously ineffective drugs against drug-resistant bacteria.

## Figures and Tables

**Table 1:** Structural data available for septal PGN hydrolases of *M. tuberculosis.*

Protein	Code	Fragment	PDB code	Ref
RipA	Rv1477	263−472	3ne0,4q4g, 4q4n,4q4t	[20,49]
		40−240	6ewy	[50]
RipB	Rv1478	30−241	3pbi	[51]
RipD	Rv1566c	38−169	4jxb	[52]
		38−182	4lj1	[52]
RpfB	Rv1009	194−362	3eo5	[25]
		282−362	4emn,4kl7,4kpm	[23,53]
		115−362	5e27	[24]
RpfC	Rv1884c	68−159	4ow1	[54]
		68−146	2n5z	[55]
RpfE	Rv2450c	98−172	4cge	[56]

## Abbreviations

AG	arabinogalactans
AnhydroGMDP	*N*-acetylglucosaminyl-β(1→4)-N-acetyl-1,6-anhydromuramoyl-l-alanyl-d-isoglutamate
CHAP	Cys, His, Asp Peptidase
DAP	meso-diaminopimelic acid
Fts	filamenting temperature-sensitive
GlcNAc	β−(1-4)-linked-*N*-acetylglucosamine
LM	lipomannan
LAM	lipoarabinomannan
MA	mycolic acids
MurNAc	N-acetylmuramic acid
PDB	Protein Databank
PGN	peptidoglycan
PIMs	phosphatidylinositol mannosides
Rip	resuscitation-promoting factor interacting protein
Rpf	resuscitation-promoting factor
SP	signal peptide
STPK	Serine/Threonine Protein Kinase
TAT	Twin-Arginine Translocation
